# Time and Covid-19 stress in the lockdown situation: Time free, «Dying» of boredom and sadness

**DOI:** 10.1371/journal.pone.0236465

**Published:** 2020-08-10

**Authors:** Sylvie Droit-Volet, Sandrine Gil, Natalia Martinelli, Nicolas Andant, Maélys Clinchamps, Lénise Parreira, Karine Rouffiac, Michael Dambrun, Pascal Huguet, Benoît Dubuis, Bruno Pereira, COVISTRESS network, Jean-Baptiste Bouillon, Frédéric Dutheil

**Affiliations:** 1 Université Clermont Auvergne, CNRS, LAPSCO, Clermont-Ferrand, France; 2 Université de Poitiers, CNRS, UMR 7295, Poitiers, France; 3 University Hospital of Clermont Ferrand, CHU Clermont–Ferrand, Clinical Research and Innovation Direction - Biostatistics, Clermont-Ferrand, France; 4 University Hospital of Clermont Ferrand, CHU Clermont–Ferrand, Occupational and Environmental Medicine, Clermont-Ferrand, France; 5 Université de Genève, UNIGE, Fondation INARTIS, Genève, Switzerland; 6 Université Clermont Auvergne, CNRS, LAPSCO, Physiological and Psychosocial Stress, University Hospital of Clermont–Ferrand, CHU Clermont–Ferrand, Emergency Medicine, Clermont–Ferrand, France; 7 Université Clermont Auvergne, CNRS, LAPSCO, Physiological and Psychosocial Stress, University Hospital of Clermont–Ferrand, CHU Clermont–Ferrand, Occupational and Environmental Medicine, WittyFit, Clermont–Ferrand, France; Tilburg University, NETHERLANDS

## Abstract

A lockdown of people has been used as an efficient public health measure to fight against the exponential spread of the coronavirus disease (Covid-19) and allows the health system to manage the number of patients. The aim of this study (clinicaltrials.gov NCT 0430818) was to evaluate the impact of both perceived stress aroused by Covid-19 and of emotions triggered by the lockdown situation on the individual experience of time. A large sample of the French population responded to a survey on their experience of the passage of time during the lockdown compared to before the lockdown. The perceived stress resulting from Covid-19 and stress at work and home were also assessed, as were the emotions felt. The results showed that people have experienced a slowing down of time during the lockdown. This time experience was not explained by the levels of perceived stress or anxiety, although these were considerable, but rather by the increase in boredom and sadness felt in the lockdown situation. The increased anger and fear of death only explained a small part of variance in the time judgment. The conscious experience of time therefore reflected the psychological difficulties experienced during lockdown and was not related to their perceived level of stress or anxiety.

## Introduction

In 2020, faced with a virus that is uncontrollable because of its unknown [1] and virulent nature (SARS-CoV-2), the governments of different countries of the European Union, as well as of the whole world, found themselves obliged to impose a lockdown on their citizens. This unprecedented public measure is thought to allow the health system to manage the number of patients in hospital and ensure that they receive proper care in the context of the Covid-19 outbreak. In France, confinement was officially imposed in the month of March (on March 17^th^ at 12:00 noon). This lockdown, which requires a large number of people to stay at home, thus depriving them of their liberty, is a situation never previously encountered and its psychological consequences in the short and medium term are not yet known. Researchers into time perception can nevertheless easily imagine that this life in lockdown completely changes individuals’ relationship to time, i.e. their experience of time. However, to our knowledge, no studies have as yet investigated this question. Very recent scale surveys or survey projects on Covid-19 conducted all around the word (e.g., China, Korea, Iran and United Kingdom) suggest that the lockdown situation generates new or heightened emotional states in the form of an increase in psychological distress [2–6]. Nonetheless, in the different distress scales used, the different dimensions of emotion (valence and arousal) were not dissociated, and no survey has examined their relationships to time experience, even though emotion and the experience of time are known to be intrinsically linked. The aim of the present study was thus to conduct a scale survey on a large sample of an as yet untested population—French people—in order to assess not only the perceived stress related to Covid-19 but also the emotions (happiness, boredom, arousal) felt during as compared to before the lockdown and their links to the subjective experience of time.

The experience of time corresponds to one’s feeling about time, i.e., the conscious judgment of the speed of the passage of time [7,8]. This has received relatively little attention by researchers in the field when compared to research into individuals’ abilities to perceive short durations (< 1 minute). This is probably due to the challenge of objectively examining just what makes up the experience of each individual, and therefore the role of higher-level cognitive mechanisms (e.g., consciousness, memory, self-awareness) [9–11]. Indeed, the judgment of the passage of time can be seen as a mirror of the subjective experience of one’s internal state [12–14]. For example, contrary to the generally held belief that time seems to pass faster as we get older, some studies have demonstrated that the feeling of the passage of time in the immediate moment is not directly related to age (young adult vs. older adult), but to people’s subjective emotional experience and lived activities [10,15,16]. The passage of time is in fact a sensitive index of emotional experience felt in the present moment and of its variations as a function of life conditions. It is thus important to investigate individuals’ judgments about how fast time seems to pass in the exceptional situation of lockdown and the factors explaining these.

From a general standpoint, the literature provides evidence of the role of emotional experience as a critical factor in the experience of time. Nevertheless, the famous expression "time flies when you feel good; time drags when you feel bad" is not straightforward to explain, as negative feelings are diverse and may involve varying mechanisms. More precisely, the emotional experience can be divided into two fundamental dimensions, valence (pleasure vs. displeasure) and activation (calmness vs. excitement/alertness) [17,18]. These two dimensions interact in the characterization of any given emotion. For example, while the emotions of sadness and fear are both negative, the former is weakly activating (or even deactivating) while the latter is strongly activating. Accordingly, the level of felt arousal has been shown to be a prominent factor in temporal mechanisms: The more individuals report being in a state of arousal, the faster time is reported to pass. Several studies have shown a lengthening of estimates of short temporal intervals in situations of acute stress, for example when participants are faced with unpleasant stimuli [19–21] or when they imminently expect a very unpleasant event, e.g., electric shock [22,23]. However, few studies have examined the effect of chronic stress on time judgments, such as that experienced by people with the Covid-19 virus or subjected to lockdown. In the context of chronic stress, i.e. when stress is extended over several days or weeks as in the case of hospital nurses, Cocenas-Silva et al. [24] showed that duration judgments were no longer altered by physiological stress as measured by physiological markers, but rather by subjective psychological stress as assessed by a self-reported scale. In addition, one can assume that different mechanisms are at work in the case of an emotion, such as fear (an immediate and ephemeral negative state directed towards a specific event), compared to a more diffuse affective state, like anxiety or perceived stress (a prolonged negative state whose origin is not necessarily identified) [25].

The Covid-19 pandemic, i.e., the risk that you or your loved ones will be affected by the disease as well as uncertainty about this disease, could produce chronic stress that has consequences for mental and physical health. It is well known that chronic stress affects the immune system, suppressing protective and increasing pathological immune responses [26]. There is thus a risk in this period of pandemic that the chronic stress related to Covid-19 and its corollaries (anxiety, fear of death) are particularly high and therefore impact the subjective experience of time by speeding up the perceived passage of time. Consequently, we hypothesized a significant relationship between stress and time experience during the lockdown imposed by the Covid-19 pandemic.

Furthermore, in this Covid-19 period, it is critical to consider not only the disease-related perceived stress but also the consequences for life of being locked down at home, as well as the direct and indirect effects on daily psychological and social functioning. As a recent survey highlighted, confining people increases their sense of boredom [2]. Boredom corresponds to “The aversive state of wanting, but being unable, to engage in satisfying activity” and involves, in particular, low arousal, negative affects [27, p 483]. In particular, some studies have shown that boredom produces a feeling of the slowing down of time rather than a speeding up [14,28]. An alternative hypothesis was thus that boredom would prevail over stress in the experience of time. Since boredom is associated with negative emotion of low level of arousal, we thus expected participants to experience of slowing down of time with the boredom experienced during the lockdown.

It was not possible *a priori* to identify which hypothesis would be valid, i.e., which are the factors related to and influencing the experience of time in a lockdown situation, the perceived stress in the stressful situation of Covid-19 and/or–by contrast–other affective states characterized by a decrease in arousal such as boredom. Indeed, on one hand, the fear and distress generated by the morbid nature of the crisis and its repercussions (fear for one’s health and for that of one’s family and friends) or by inappropriate housing quality (stress at home) or working conditions (job stress) could increase people’s sense of alertness, and therefore lead to a speeding of the passage of time. On the other, confinement at home and social distancing could result in an increased sense of sadness (i.e., less happiness) and boredom, and thus in the feeling that the passage of time slows down. Here, a large sample of French people were asked to answer a scale survey during the lockdown period. This consisted of a series of questions, i.e., demographic questions but also questions on the stress perceived (Covid-19 stress, home stress, job stress, anxiety), the emotions (happiness, arousal, boredom) felt during compared to before the lockdown and the experience of time. The participants were asked to assess their experience of the passage of time according to three periods of the lockdown: in the immediate moment, during the day, during the last week, as well as before the lockdown for comparison purposes.

## Method

### Participants

The sample consisted of 4364 French participants, 3436 women and 928 men (Mean age = 41.5, SD = 12.81, Min = 16, Maxi = 89, N_16-17 years_ = 11). The participants completed the questionnaire at home (72.5%) or at work (27.5%). The study was reviewed and approved by the human ethics committees Sud Est VI, France (clinicaltrials.gov NCT 04308187). All participants were volunteers and were informed of the objective of the survey and that their data would be processed anonymously and be used for research purposes. The ethics committee waived the need for written consent considering that if people respond to the questionnaires by going to the website, they are giving their consent. Furthermore, they can withdraw it at any time. The few minors who completed the questionnaire did so with the consent of their parents who sent them the survey.

The responses to the demographic questions allowed us to characterize the surveyed population. 71.8% of participants were married or equivalent (civil partner, etc.) and 27.2% were single (1% other). Their distribution as a function of education level was: 1.5% certificate of general education, 21.9% high school vocational certificate, 0% high school diploma, 40.6% bachelor’s degree, 24.5% master’s degree and 11% doctoral degree. The percentage of participants per professional category was: Jobseekers: 4.4%; Students: 6.2%; Farmers: 0.3%; Craftsmen/shopkeepers/business executives: 5.7%; White-collar workers: 30%; Manual workers: 8.9%; Intermediate professions, 35.7%; Retired: 6.3% (2.5% no response).

### Procedure

We implemented an open epidemiological, observational, descriptive study by administering a self-reported questionnaire proposed to volunteers using REDCap^®^ software available through the COVISTRESS.ORG website. The REDCap^®^ questionnaire was hosted by the University Hospital of Clermont-Ferrand. The questions analyzed in this manuscript were therefore specific questions included in a large questionnaire composed of different thematic sections of questions (S1 Questions). The thematic sections were presented in random order after the demographic questions. The online questionnaire was distributed several times through mailing lists held by institutions and French social groups. There were no exclusion criteria. The data that we analyzed were obtained for the period of lockdown from March 31^th^ to April 12^th^, 2020, whereas the French lockdown was ordered on March 17^th^ at 12:00 noon. The time taken to complete the survey lasted between 5 and 20 minutes on average, depending on sub-items.

For the main outcomes, we used a visual analog scale (VAS), i.e., a non-calibrated line of 100 mm, ranging from 0 to 100 [29,30]. The subjective experience of time was thus assessed using this VAS, which went from very slowly (0) to very fast (100). The question was “what are your feelings about the speed of the passage of time”. There were four time questions, one for the passage of time before the lockdown, and three for during the lockdown: now, for the day, and for the week. The stress resulting from Covid-19 as well as job stress and home stress, health-related and financial concerns and anxiety were assessed using the same VAS. The emotional dimensions tested were also assessed with the VAS for the period before the lockdown and during the lockdown (now): fear of death (not at all *vs*. at lot), arousal (calm *vs*. excited), happiness (sad *vs*. happy), anger (peaceful vs. angry), boredom (occupied *vs*. bored). The quality of sleep and level of fatigue were also examined in the survey using the VAS. As explained above, these different questions were presented in different thematic sections presented in a random order (S1 Questions).

### Statistical analyses

We performed analyses of variance on the subjective experience of time. We also examined correlations and ran a linear regression model on all the measures of interest by using the standardized data. We used the variance inflation factor (VIF) to examine the multicollinearity in the regression analysis [31]. Finally, to examine the results of the linear regression model in more detail, we also performed an analysis of mediation. The analyses were performed with SPSS and the Bonferroni correction was systematically applied when necessary.

## Results

### Experience of time

**Fig 1:**
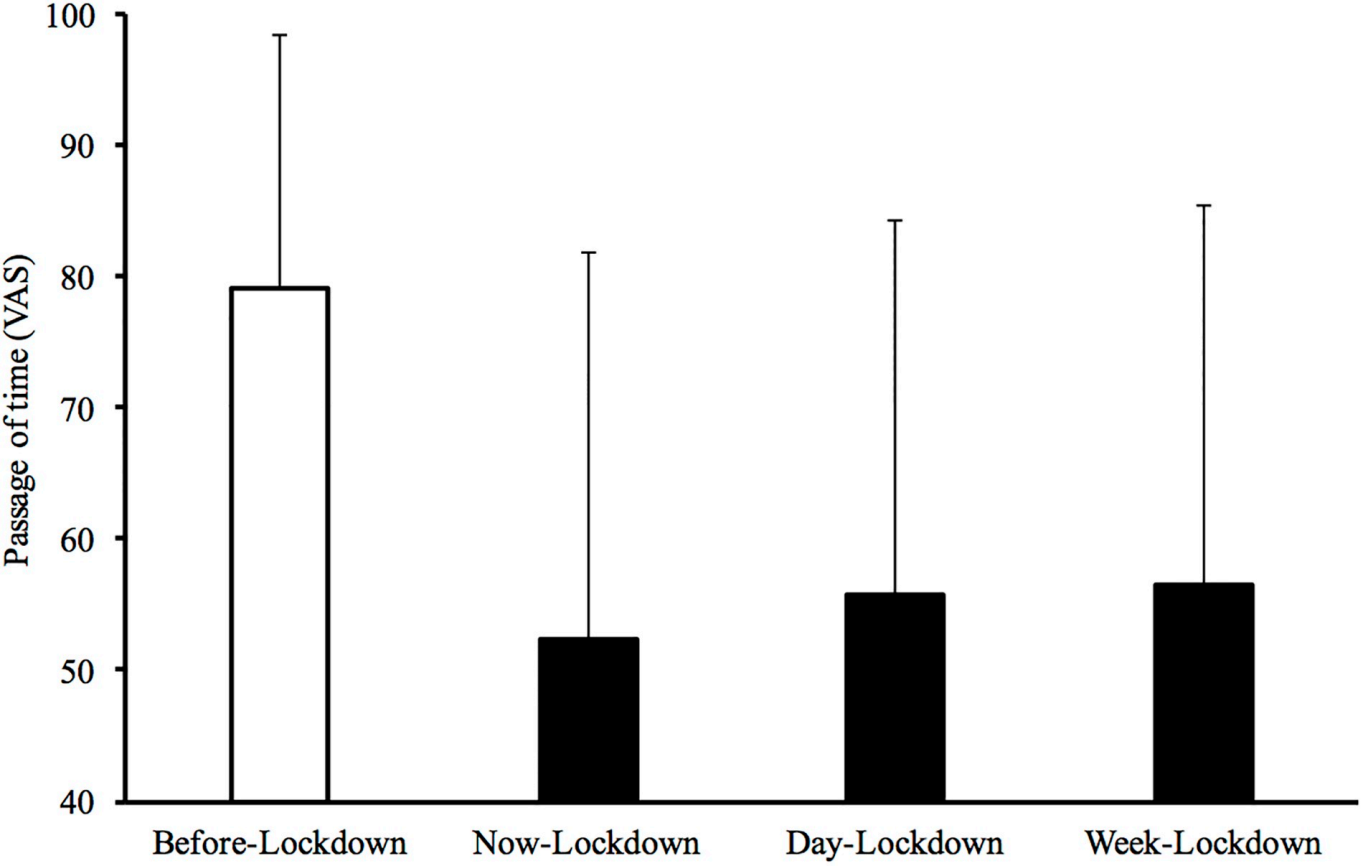
Mean passage of time for the period before the lockdown and during the lockdown, i.e., for the present, the day and the week.

A preliminary analysis of variance performed on the subjective experience of time showed a marked difference between the experience of time before and during the lockdown (Fig 1). The participants reported that time passed faster before the lockdown (M = 79.18, SD = 19.31) than during the lockdown for different time periods, i.e. in the present (M = 52.24, SD = 29.58), F(1, 4360) = 2634.85, p < 0.001, η^2^_p_ = .38; for the day (M = 55.64, SD = 28.68), F(1, 4351) = 2162.37, p < 0.001, η^2^_p_ = .33; for the week (M = 56.44, SD = 28.97), F(1, 4339) = 1946.21, p < 0.001, η^2^_p_ = .31. The difference between these three temporal periods of lockdown were small but significant, F(2, 8660) = 138.35, p < 0.001, η^2^_p_ = .03, with participants reporting that time passed faster when a longer period of time was considered, i.e., a week compared to a day or the present moment (Bonferroni comparisons, p < 0.01).

To simplify the results, the subsequent statistical analyses are based on the difference in time ratings for the question on the period before the lockdown and that for the present moment (during the lockdown). Indeed, the meaning of temporal judgment during the lockdown is relative to that before the lockdown. In addition, the results were similar when the analyses were only performed on the ratings for the present moment. A positive value of our temporal difference index therefore indicates that the individuals experience a slowing down of time during the lockdown, a negative value a speeding up of time and a null value no difference.

The ANOVA performed on this temporal difference index, with level of education, professional category and whether the individuals were at work or home as factors, did not show any significant effect (all F < 1). There was indeed no significant difference in time experience before the lockdown situation as a function of these factors. Only a small effect of professional category was observed in the present time judgment during the lockdown, F(8, 4224) = 3.62, p < 0.001, η^2^_p_ = .007 (same results for the day and the week). This was mainly due to manual workers (M = 37.69, SE = 4.88), who felt that time passes slower than the other categories (white-collar workers, M = 62.40, SE = 4.39; intermediate professions, M = 54.43, SE = 2.68; retired, M = 64.10, SE = 5.21) (Bonferroni, p < 0.05), although their time feeling was similar to that of the jobseekers (M = 39.71, SE = 39.71), students (M = 48.95, SE = 3.15), craftsmen/ shopkeepere/business executives (M = 51.62, SE = 4.23), and farmers (M = 52.23, SE = 10.07) (p > 0.05). There was no significant difference between most of the other professional activities (i.e., students, white-collar workers, intermediate professions, retired, craftsmen/ shopkeepers/business executives, farmers) (all p > 0.05).

The ANOVA on the temporal index with sex and marital status (single *vs*. not single) as factors showed a significant main effect of sex, F(1, 4084) = 14.77, p < 0.001, η^2^_p_ = .004, and status, F(1, 4084) = 11.74, p < 0.001, η^2^_p_ = .003, with no sex x status interaction (p > 0.10). This suggests that the single people in our sample tended to experience a greater difference in the flow of time during the lockdown when compared to before (29.19 *vs*. 24.19). Indeed, in the lockdown situation, time in the present was judged to pass slower by the single people (M = 47.12, SD = 30.12) than by the others (M = 53.93, SD = 29.15). The women also tended to feel a greater slowing down of time than the men (29.41 *vs*. 23.89) during as compared to before the lockdown, but time passed faster for the women than for the men before the lockdown (80.51 *vs*. 74.37), F(1, 4084) = 71.11, p < 0.001, η^2^_p_ = .02. Nevertheless, their responses to the stress questions indicated that they tended to be more stressed than the men, even though the sex difference only explained a very small proportion of variance (Covid-Stress, 64.17 *vs*. 51.95, F(1, 4195) = 129.09, p < 0.001, η^2^_p_ = .03; Home stress, 48.51 *vs*. 41.21, F(1, 4183) = 35.11, p < 0.001, η^2^_p_ = .01; Job stress, 59.75 *vs*. 51.95, F(1, 3891) = 41.95, p < 0.001, η^2^_p_ = .01).

### Correlations between experience of time, stress and other factors

Table 1 shows the correlation matrix (S1 Table) between the subjective experience of time (difference in the judgment of the passage of time between before the lockdown and the present moment, i.e., during the lockdown) and the different tested factors. An examination of Table 1 reveals that several dimensions were associated with the slowing down of time during as compared to before the lockdown. With regard to stress, the participants experienced that time passed slower—rather than faster—with an increase in the level of perceived stress, i.e., the perceived stress related to Covid-19 (R = .18) as well as the stress at home (R = .23) and at work (R = .08). A slowing down of time was therefore observed as the stress level increased. This deceleration of subjective time was observed even if the stress value reported on the VAS was high, and higher for Covid-19-related stress than for home and job stress (Covid-19 stress, M = 61.50, SD = 28.87; Job stress, M = 57.94, SD = 32.65; Home stress, M = 46.97, SD = 32.65, F(2, 7466) = 342.78, p < 0.001, η^2^_p_ = .08 (all Bonferroni tests, p < 0.001). The rating for each type of stress was indeed significantly different from zero (t(4196) = 138.18, t(4184) = 93.06, t(3892) = 10.13, respectively, all p < 0.001). Finally, the stress resulting from Covid-19 was more closely associated with anxiety (R = .75, p < 0.001), the fear of death (R = -.42, p < 0.001) than it was with the experienced time *per se*.

**Table 1:** Correlations between the passage of time (difference between before the lockdown and for the present, i.e., during the lockdown) and the different tested factors (z-scores).

	Time^a^	1	2	3	4	5	6	7	8	9	10	11	12	13
1.Age	-.07**													
2.Covid stress	.18**	.01												
3.Home stress	.23**	-.06**	.53**											
4.Job stress	.08**	-.06**	.43**	.30**										
5.Health con.	.16**	.12**	.69**	.39**	.29**									
6.Financial con.	.11**	.10**	.13**	.11**	.06**	.12**								
7.Arousal^a^	-.11**	-.03	-.16**	-.19**	-.16**	-.14**	-.02							
8.Anxiety	.20**	-.03	.75**	.54**	.42**	.64**	.10**	-.19**						
9.Fear of death^a^	-.23**	-.03	-.42**	-.26**	-.18**	-.46**	-.08**	.16**	-.42**					
10.Anger^a^	-.31**	.02	-.29**	-.30**	-.18**	-.22**	-.07**	.43**	-.30**	.28**				
11.Happiness^a^	.39**	.01	.33**	.28**	.19**	.26**	.12**	-.27**	.34**	-.33**	-.54**			
12.Boredom^a^	-.48**	.13**	-.12**	-.17**	.01	-.11**	-.06**	.06**	-.16**	.16**	.26**	-.34**		
13.Sleep	-.04*	.05**	-.07**	-.07**	-.08**	-.04*	.01	.03	-.06**	.04*	.07**	-.09**	.03*	
14.Tired	.12**	-.09**	.43**	.43**	.44**	.30**	.04*	-.20**	.45**	-.21**	-.22**	.21**	-.04*	-.14**

^a^Difference in ratings between before the lockdown and for the present (during the lockdown);

* *p* < .05,

** *p* < .01; con. = concerns.

Inconsistently with our first hypothesis, the level of correlation between the experience of time and Covid-19-related stress was therefore very low, and this was also the case for stress in the other contexts (home, work). As suggests Table 1, the experience of time was more correlated with boredom (R = -.48, p < 0.001) and decreased happiness (R = .39, p < .0001) than with the level of perceived stress. Therefore, the participants experienced a slowing down of time as boredom increased and happiness decreased during the lockdown. Although significant, the correlations between the experience of time and the other factors remained weaker (Age: R = -.07; Health concerns, R = .16; Economical concerns, R = .11; Arousal, R = -.11; Anxiety, R = .20; Fear of death, R = -.23; Anger, R = -.31; Sleep, R = -.04; Tired, R = .12).

### The best predictors of the experience of time during the lockdown

As the time judgment was significantly correlated with several dimensions, to identify the best predictor of the subjective experience of time we performed a regression analysis on the time judgments with the different significant dimensions entered into the same model (Table 2). The examination of multicollinearity in the regression analysis using the VIF indicated no problematic presence of multicollinearity (all VIF < 3) [31]. The results of this regression analysis indicated that the perceived stress resulting from Covid-19 and its spread was not a significant predictor of changes in the experience of time (p > 0.05). In line with this finding, the levels of anxiety, arousal and health concerns, which were highly correlated with stress (R = .75, R = -.16, R = .69, p < 0.001), did not explain the changes in the time experience (p > 0.05). Only the level of perceived stress at home explained a small part of the variance (B = .08, SE = .02, β = .07, t = 3.81, p < 0.001).

**Table 2:** Potential predictors of the passage of time when all factors were included in the regression model.

	B	SE	β	t	p	95% CI		VIF
						Lower	Upper	
(Constante)	0.012	0.015		0.783	0.434	-0.018	0.042	
Age	-0.002	0.017	-0.002	-0.128	0.898	-0.035	0.031	1.086
Covid stress	-0.04	0.026	-0.04	-1.499	0.134	-0.092	0.012	2.928
Home stress	0.074	0.019	0.074	3.812	0.001	0.036	0.113	1.562
Work stress	0.007	0.018	0.007	0.365	0.715	-0.029	0.042	1.371
Health concern	0.002	0.023	0.002	0.093	0.926	-0.043	0.047	2.182
Eco. concern	0.055	0.015	0.056	3.566	0.001	0.025	0.085	1.038
Arousal	0.029	0.018	0.028	1.63	0.103	-0.006	0.063	1.239
Anxiety	0.001	0.025	0.001	-0.008	0.993	-0.049	0.049	2.598
Death fear	-0.07	0.018	-0.07	-3.842	0.001	-0.106	-0.034	1.375
Anger	-0.092	0.02	-0.09	-4.564	0.001	-0.132	-0.052	1.631
Happiness	0.177	0.02	0.174	8.866	0.001	0.138	0.216	1.613
Boredom	-0.375	0.017	-0.372	-21.884	0.001	-0.408	-0.341	1.203
Sleep	-0.006	0.016	-0.006	-0.369	0.712	-0.037	0.025	1.027
Tired	0.028	0.019	0.028	1.484	0.138	-0.009	0.066	1.499

The fear of death and the emotion of anger expressed by the participants during the lockdown were also higher when compared to the situation before the lockdown, even if the level of fear of death and anger remained low (< 50) (Fear of death, 46.11 *vs*. 30.41, F(1, 3647) = 1557.50, p < 0.001, η^2^_p_ = .30; Anger, 56.19 vs. 38.49, F(1, 3621) = 1181.76, p < 0.001, η^2^_p_ = .25). Therefore, the fear of death and anger explained a proportion of variance in changes in time judgment (B = -.07, SE = .02, β = -.07, t = -3.84; B = -.10, SE = .02, β = -.09, t = -4.56, respectively, both p < 0.001). The more afraid people were of death and the angrier they felt, the more time seemed to drag. However, the proportion of variance explained was also very low.

**Fig 2:**
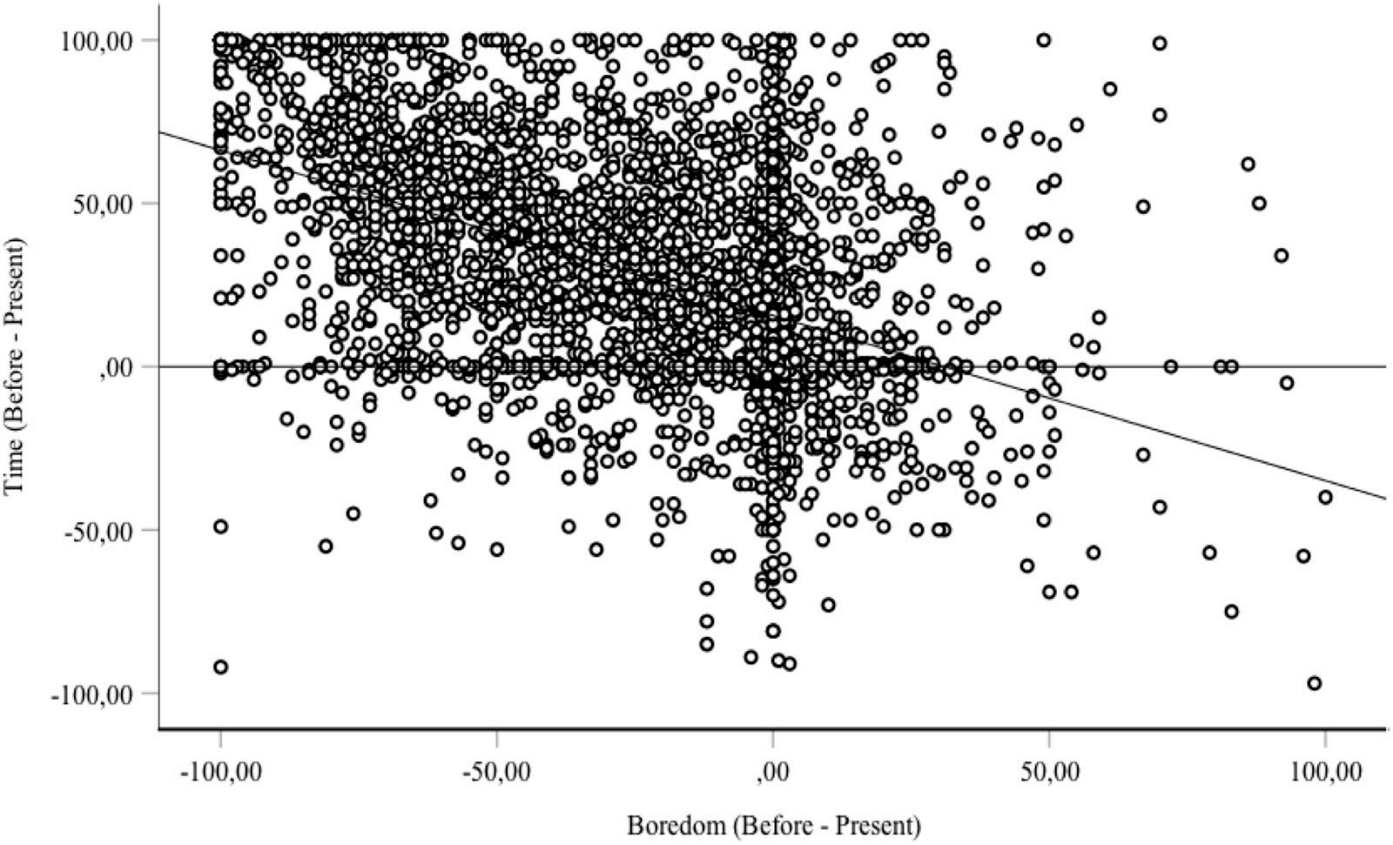
Relationship between passage of time and boredom (difference in judgments before the lockdown and during the lockdown, i.e., for the present).

**Fig 3:**
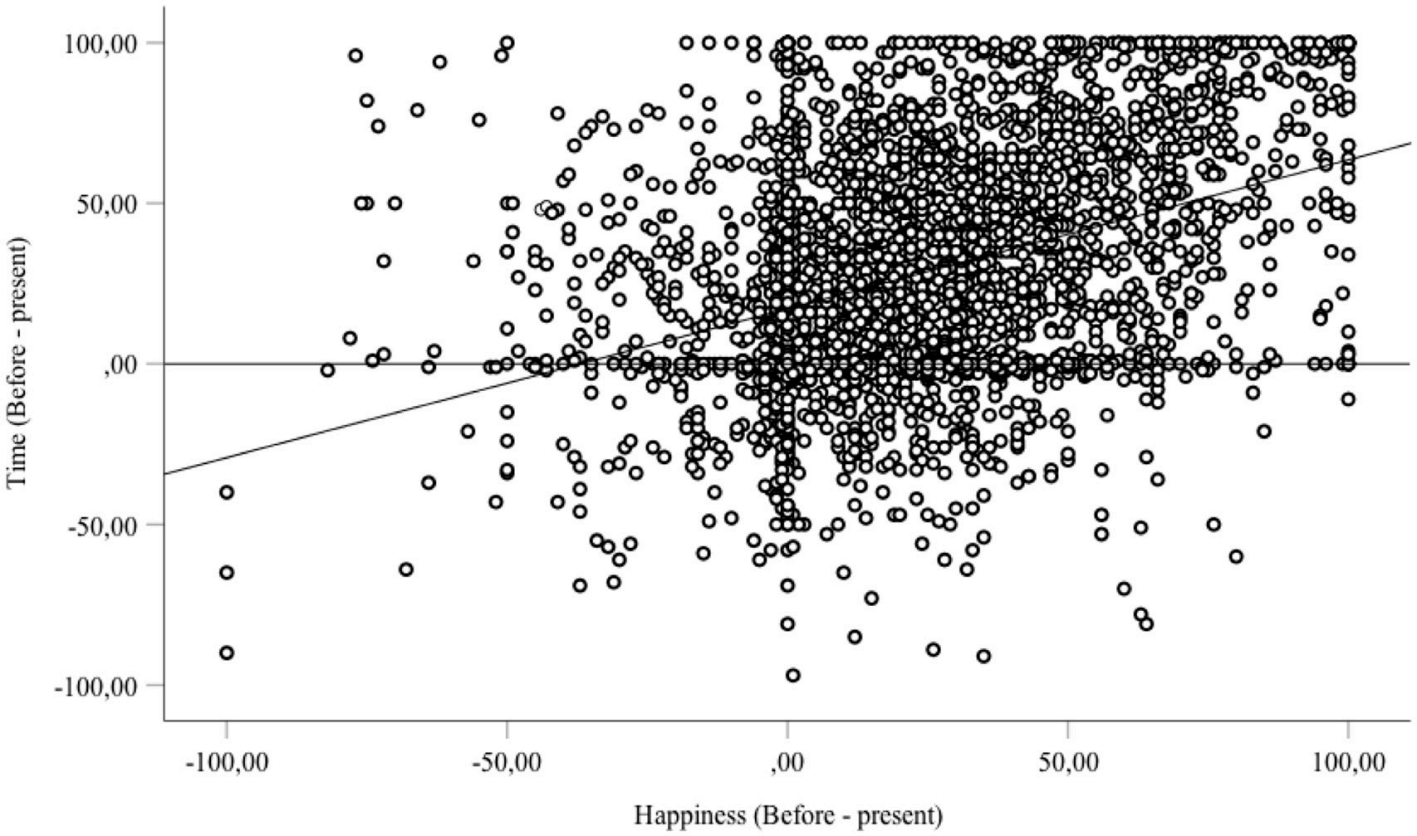
Relationship between passage of time and emotion (sadness *vs*. happiness) (difference in judgments before the lockdown and during the lockdown, i.e., for the present).

The most reliable predictor of time experience in the lockdown situation was the boredom felt during this period (B = -.38, SE = .017, β = -.37, t = -21.88, p < 0.001), and the emotion of sadness (B = .18, SE = .02, β = .17, t = 8.87, p < .0001). Hierarchical regression analysis with the boredom and the emotion of sadness, and the other factors entered step-by-step into the equation revealed that the added part of variance explained by these factors remained very small (e.g., Δ < .01 for home stress, anger, fear of death). Therefore, people felt more bored during the lockdown than before it (41.48 *vs*. 18.60, F(1, 4112) = 1968.67, p < 0.001, η^2^_p_ = .32), and they also felt less happy (sadder) (46.04 *vs*. 68.31, F(1, 3903) = 2281.19, p < 0.001, η^2^_p_ = .37). In the survey, boredom and the emotion felt were obviously correlated (R = -34, p < 0.001). Boredom was not correlated with the arousal level (R = .06, p > 0.05) and was only weakly correlated with anxiety (R = .16, p < 0.01). Indeed, the level of arousal increased only slightly during the lockdown period compared to before (47.55 *vs*. 44.22, F(1, 3857) = 45.41, p < 0.001, η^2^_p_ = .01). Consequently, the more bored and less happy the participants were in the lockdown situation, the more they experienced a slowing down of time. Indeed, time was experienced as passing increasingly slowly in the present moment compared to before the lockdown as the level of boredom rose (Fig 2). It also seemed to slow down as happiness decreased, i.e., as sadness increased (Fig 3).

### Mediation analyses

**Fig 4:**
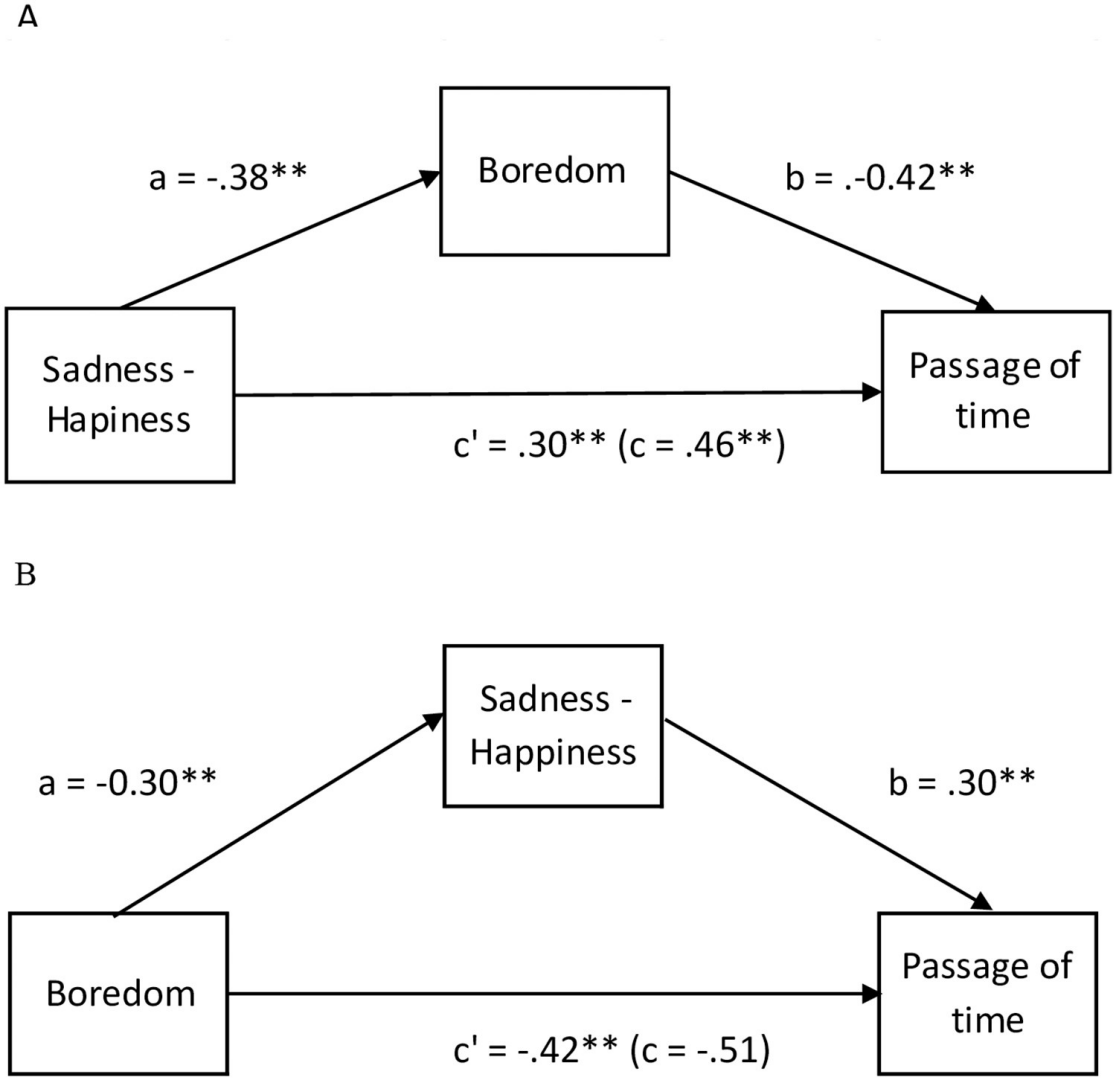
Mediation models with the boredom as mediator of the effect of the emotion (sadness *vs*. happiness) on the experience of the passage of time (A) and with the emotion as mediator of the effect of the boredom on the experience of the passage of time (B).

Increasing boredom and decreasing happiness were therefore the two main predictors of the experience of the passage of time during the lockdown. Since these two dimensions are related, we conducted statistical analyses to estimate whether the boredom mediated the effect of emotion on the experience of time and, conversely, whether emotion mediated the effect of the boredom of the experience of time. The mediation analyses indicated that boredom contributes to explaining the effect of emotion on the experience of the passage of time, with a significant indirect effect of 0.159 (β), SE = .01, 95% CI (.138; .1812), Z = 14.7, p < 0.001, 34.4% of mediation) (Fig 4). However, the direct effect of emotion (sadness) on the time experience remained significant (β = .30, ES = .02, 95%CI[.26; .346], Z = 14.3, *p* < 0.001, 65.6% of mediation). The emotion also contributed to explaining the effect boredom on the experience of the passage of time, with a significant but small indirect effect of -0.08 (β), SE = .008, 95%CI [-.106; -.078], Z = 12.0, p < 0.01, 17.9% of mediation), but the direct effect of boredom remained obviously significant (p < 0.001). Therefore, boredom was not the only factor explaining the experience of time in lockdown. In conclusion, the temporal judgment reflected the boredom and greater level of sadness of people living in lockdown and was not related to the stress or anxiety induced by Covid-19.

## Discussion

The results of our survey showed that the stress felt by a broad cross-section of the French population during the lockdown was high, in particular with regard to stress relating to the Covid-19 pandemic, as is indicated by the rating of 61.50 (+/-28.87) on a 100-mm VAS. The level of perceived stress linked to Covid-19 was even higher than the stress at work and at home. Covid-19 stress was, in fact, related to the participants’ anxiety and their fear of death. The more anxious and frightened they were about death, the more stressed they were in the face of this disease. These results are entirely consistent with the initial results of surveys on Covid-19 conducted, in particular, in China [5,6] and Iran [4], which have shown an increase in psychological distress as a result of the Covid-19 pandemic. However, as reported by Qui et al. [5], it is noteworthy that people’s distress does not reach a pathological level (M = 23.65), with only 5% of the population suffering from severe distress and 29% from mild or moderate distress. In addition, the proportion of individuals presenting psychological distress disorders before the Covid-19 is unknown. However, the Chinese suffer less psychological distress and have greater life satisfaction when working in the office than at home, whereas the opposite seems to be the case in the French population, as suggested by the significantly lower level of stress at home than at work. This suggests that there are some differences in culture or living conditions between people in different countries with regard to stress management in similar social isolation situations.

The originality of our results is to show that, although the level of stress was quite high, it had little impact on the current subjective experience of time. Indeed, the participants did not feel a speeding up of time related to the increase in their stress level. This is contrary to the results of studies on timing which have described a lengthening of duration estimates and the experience of a faster passage of time when the levels of stress and anxiety are high [21,32,33]. However, these findings were obtained in intense and concisely emotional situations, when the subjects were faced or expecting a forthcoming threatening event, or in individuals with high-anxiety traits. In the situation of lockdown at home, the current level of stress was therefore not high enough to affect the sense of time. Indeed, the level of arousal remained low, although it increased slightly between the period before and during the lockdown. To conclude, one might nevertheless think that it would have been more convincing to record the physiological markers of stress. However, this was not possible in the lockdown situation which was rapidly decided on by the public authorities [34,35]. In addition, Cocenas et al. [24] recently showed that perceived stress was a better predictor of changes in time estimates than physiological stress *per se* in the case of prolonged stressful situations, for example in the case of hospital nurses at work. In addition, the likelihood of encountering a series of intensely stressful events may be reduced in the present isolation situation. Family life involving the care of children can obviously be a source of stress. Our study did indeed indicate that women were more stressed at home than men, but were even more so when they were single than part of a family, and that the number of children only slightly increased the stress level at home (R = .08, p < 0.001).

Rather than Covid-19-related stress or home and job stress, our study showed that it was the emotional experience of everyday life during the lockdown that influenced the sense of time. Indeed, the participants clearly reported experiencing a slowing down of the passage of time during in comparison to before the lockdown. And the most reliable predictors of this slowing down were the feelings of boredom and sadness. Our results are consistent with those of recent studies on time judgments that have pointed out the critical role of emotion in human beings’ sense of time [for a review 35] and of boredom [14,28,36]. These studies have indeed found a slowing down of time as both sadness and boredom increase. In line with theoretical models of boredom [27], the present study found that the degree of boredom experienced was related not only to arousal but mostly to negative emotional experience: The more bored people were in lockdown, the sadder they were. The boredom is known to be linked to depression [37,38], and depressed people feel a slowing down of time [39]. Consequently, the experience of boredom in the lockdown and the judgment of a slower passage of time have increased sadness and could lead to pathological depression. However, in the lockdown situation, the level of boredom explained a proportion, but not all, of the effect of sadness on the experience of the passage of time. Other factors that we need to examine in a future study could also help to explain sadness and time experience in the lockdown, such as social withdrawal.

The changes in the sense of time in lockdown were therefore due to the significant increase in both boredom and sadness. The literature on boredom suggests that it is involved in a multitude of behaviors and psychological dimensions and that it has a negative side, as in the sadness observed in our study, as well as a positive side. Indeed, trait boredom is associated with psychological difficulties (e.g., drug abuse, depression, anxiety, binge eating) [40,41]. However, some recent functional approaches have also suggested that boredom constitutes a key signal to change behavior by orientating humans to try to find a more satisfying situation [42]. In the context of lockdown, one may therefore wonder what influence this feeling of boredom has on the development of pro-social behaviors or on compliance with the containment situation in the short or longer term (does it only result in bad things or also in good things?).

In the lockdown situation, people may have more time. However, they “die” of boredom and sadness and time slows down, drags on. The sense of the passage of time is, ultimately, a phenomenological time that is closely related to the self and the sense of existence [13]. As stated by Jean-Paul Sartre, human beings are defined by their acts and their effects on others. However, when they have more time but are isolated and cannot act—they have nothing to do—they are overwhelmed by sadness and boredom. It would seem important for future surveys to examine whether this feeling is valid in all cultures and for all people. It also seems to be important to identify whether other factors specific to individual characteristics or living conditions, to representations/beliefs toward Covid-19 or government policies contribute to changes in the sense of time in the lockdown situation. Some authors nevertheless defend the benefits of boredom. However, this raises the question of individual abilities to cope with the feeling of boredom in industrial societies. Individual differences in coping with boredom can potentially predict psychological difficulties, health problems and increased vulnerability to psychopathologies such as depression [43]. It is thus a serious problem and one which has to be taken into account. In conclusion, the changes in the sense of time in the lockdown situation, imposed as an efficient solution to the Covid-19 pandemic, reflect the major psychological difficulties that people are experiencing during the lockdown.

## Supporting information

S1 Questions Questions used from the Covistress survey (COVISTRESS.ORG website). (DOCX)

S1 Table Table of data used and analyzed in the study. (XLSX)
