# Supplementary material for: Time and Covid-19 stress in the lockdown situation: Time free, «Dying» of boredom and sadness
Source: PLoS One. 2020 Aug 10;15(8):e0236465. doi: 10.1371/journal.pone.0236465 (PMC7416923; doi:10.1371/journal.pone.0236465)
Supplement: S1 Questions — (DOCX) [file pone.0236465.s001.docx]

**Questions (with their numbers) from the sections of the questionnaire used in the French survey on stress and Covid-19 (COVISTRESS.org) that were analyzed in the manuscript published by Droit-Volet et al. (2020). Time and Covid-19 stress in the lockdown situation: Time Free, « Dying » of Boredom and Sadness. PlosOne-D-20-12336**

**Section – Epidemiological context**

**1. In which country do you work?**

**2. Gender** □ Female □ Male

**3. Age**

**4. Right now you are:** □At the emergency department □ Hospitalized □At a medical appointment □At work □At home □ In an outdoor environment □ Other

**Section - Stress and worries**

**12. What is your level of...?** VAS 0 to 100, Min | Max

... worries for your health because of Coronavirus

... stress due to Coronavirus

... stress at home

... stress at work

... fatigue

... sleep quality

... anxiety/ fear

**Section - Occupation**

**16. Actual status:**

□ **Executive and superior intellectual occupation**

□ **Intermediary profession**

□ **Farmer**

□ **Artisan, merchant or entrepreneur**

□ **Worker**

□ **Student**

□ **Looking for a job**

□ **Retired**

**17. What is your education level?**

□ GCSE or under □ youth training/BTEC □ High school graduation □ 1st to 2nd year university level □ 5th year university level: Master 2, MDE, DHS or DHAS □ over 5th year of university level: Doctorate or other

**22. What is your level of ...** VAS 0 to 100, Min | Max

... worries for economic impact because of Coronavirus

**Section - Parenthood**

**25. Marital status**: single, married, cohabitation, widowed, other

**26. Total number of children**:

**Section – Isolation and impact of Coronavirus**

**38. How do you feel about the passage of time?**

VAS « time seems to go slowly» to « time flies very fast »

... before Coronavirus epidemics

... now

... during the day

... during the week

**39. Your emotional feelings... ?**

... Soothed / Anger (VAS Soothed / Anger) Before Coronavirus epidemics

... Soothed / Anger (VAS Soothed / Anger) Now

... Sadness / Joy (VAS Sadness / Joy) Before Coronavirus epidemics

... Sadness / Joy (VAS Sadness / Joy) Now

... Peaceful / Excited (VAS Peaceful / Excited) Before Coronavirus epidemics

... Peaceful / Excited (VAS Peaceful / Excited) Now

... Busy / Boredom (VAS Busy / Boredom) Before Coronavirus epidemics

... Busy / Boredom (VAS Busy / Boredom) Now

**41. Fear of death**

… Before Coronavirus epidemics: VAS min max

… Now: VAS min max
